# Fully automated detection and localization of clinically significant prostate cancer on MR images using a cascaded convolutional neural network

**DOI:** 10.3389/fonc.2022.958065

**Published:** 2022-09-29

**Authors:** Lina Zhu, Ge Gao, Yi Zhu, Chao Han, Xiang Liu, Derun Li, Weipeng Liu, Xiangpeng Wang, Jingyuan Zhang, Xiaodong Zhang, Xiaoying Wang

**Affiliations:** ^1^ Department of Radiology, The First Affiliated Hospital of Zhengzhou University, Zhengzhou, China; ^2^ Department of Radiology, Peking University First Hospital, Beijing, China; ^3^ Department of Clinical & Technical Support, Philips Healthcare, Beijing, China; ^4^ Department of Urology, Peking University First Hospital, Beijing, China; ^5^ Department of Development and Research, Beijing Smart Tree Medical Technology Co. Ltd., Beijing, China

**Keywords:** deep learning, prostatic neoplasms, magnetic resonance imaging, detection, localization

## Abstract

**Purpose:**

To develop a cascaded deep learning model trained with apparent diffusion coefficient (ADC) and T2-weighted imaging (T2WI) for fully automated detection and localization of clinically significant prostate cancer (csPCa).

**Methods:**

This retrospective study included 347 consecutive patients (235 csPCa, 112 non-csPCa) with high-quality prostate MRI data, which were randomly selected for training, validation, and testing. The ground truth was obtained using manual csPCa lesion segmentation, according to pathological results. The proposed cascaded model based on Res-UNet takes prostate MR images (T2WI+ADC or only ADC) as inputs and automatically segments the whole prostate gland, the anatomic zones, and the csPCa region step by step. The performance of the models was evaluated and compared with PI-RADS (version 2.1) assessment using sensitivity, specificity, accuracy, and Dice similarity coefficient (DSC) in the held-out test set.

**Results:**

In the test set, the per-lesion sensitivity of the biparametric (ADC + T2WI) model, ADC model, and PI-RADS assessment were 95.5% (84/88), 94.3% (83/88), and 94.3% (83/88) respectively (all p > 0.05). Additionally, the mean DSC based on the csPCa lesions were 0.64 ± 0.24 and 0.66 ± 0.23 for the biparametric model and ADC model, respectively. The sensitivity, specificity, and accuracy of the biparametric model were 95.6% (108/113), 91.5% (665/727), and 92.0% (773/840) based on sextant, and were 98.6% (68/69), 64.8% (46/71), and 81.4% (114/140) based on patients. The biparametric model had a similar performance to PI-RADS assessment (p > 0.05) and had higher specificity than the ADC model (86.8% [631/727], p< 0.001) based on sextant.

**Conclusion:**

The cascaded deep learning model trained with ADC and T2WI achieves good performance for automated csPCa detection and localization.

## Introduction

Prostate cancer (PCa) is one of the most common malignant tumors in men worldwide. The clinical behavior of PCa ranges from low-grade indolent that is generally considered to be non-life-threatening to high-grade aggressive tumors with a Gleason of Score 7–10, i.e. clinically significant PCa (csPCa), that may progress rapidly to metastatic disease and may be life-threatening (1). Multiparametric magnetic resonance imaging (mpMRI) has adopted an increasingly significant role in the detection and localization of csPCa, as well as in guiding targeted biopsy (2). Recent large-scale clinical trials have demonstrated that the use of mpMRI before biopsy increases the detection of csPCa, while reducing the detection of those deemed clinically insignificant (3, 4). Furthermore, using mpMRI to triage male patients may enable a quarter to half of them to avoid the need for biopsy (3, 5). To standardize and improve the interpretation of prostate mpMRI, the use of the Prostate Imaging Reporting and Data System (PI-RADS) has been recommended and updated (2, 6). However, the interobserver agreement for subjective evaluation using PI-RADS (version 2) is moderate and influenced by the readers’ experience (7, 8). Additionally, PI-RADS (version 2.1) has shown no significant improvements in overall diagnostic performance compared to PI-RADS (version 2.0) (9, 10). As there is arguably a trend in more people with clinically suspected csPCa undergoing prostate mpMRI, it is clinically desirable to develop more accurate and automated methods for prostate mpMRI interpretation.

In the recent years, artificial intelligence (AI) methods, particularly deep learning, have achieved promising results in automated csPCa diagnosis of mpMRI (11–14). A range of proposed deep learning algorithms were trained based on prior annotated regions of interest (ROIs) to classify them as cancerous or noncancerous lesions (11, 15), or slices, in which each individual image was classified as cancerous or not (16, 17). These methods were unable to precisely detect and locate csPCa, and such predicted results may not be directly applied to clinical practice. Some computer-aided diagnosis (CAD) systems developed for csPCa were based on the manual or semi-automatic segmentation of the prostate gland (13, 18), which also limits their direct clinical use. With the development of the deep convolutional neural network (CNN), some approaches for csPCa detection have been fully automatic with an area under the receiver operator characteristics curve of 0.75–0.86 (12, 19, 20). More studies are needed to improve and optimize these models. Although many generalized AI models have been developed, few studies have reported on how to integrate AI-based prediction into the clinical workflow. More explorations are demanded to move the prostate AI systems from the laboratory to the clinic with perfect output.

Our study aimed to develop a fully automated cascaded deep learning model for the detection and localization of csPCa using apparent diffusion coefficient (ADC) maps and T2-weighted imaging (T2WI), as well as to seamlessly integrate these AI predictions into the radiological workflow using the structured report.

## Materials and methods

### Study subjects

**Figure 1:**
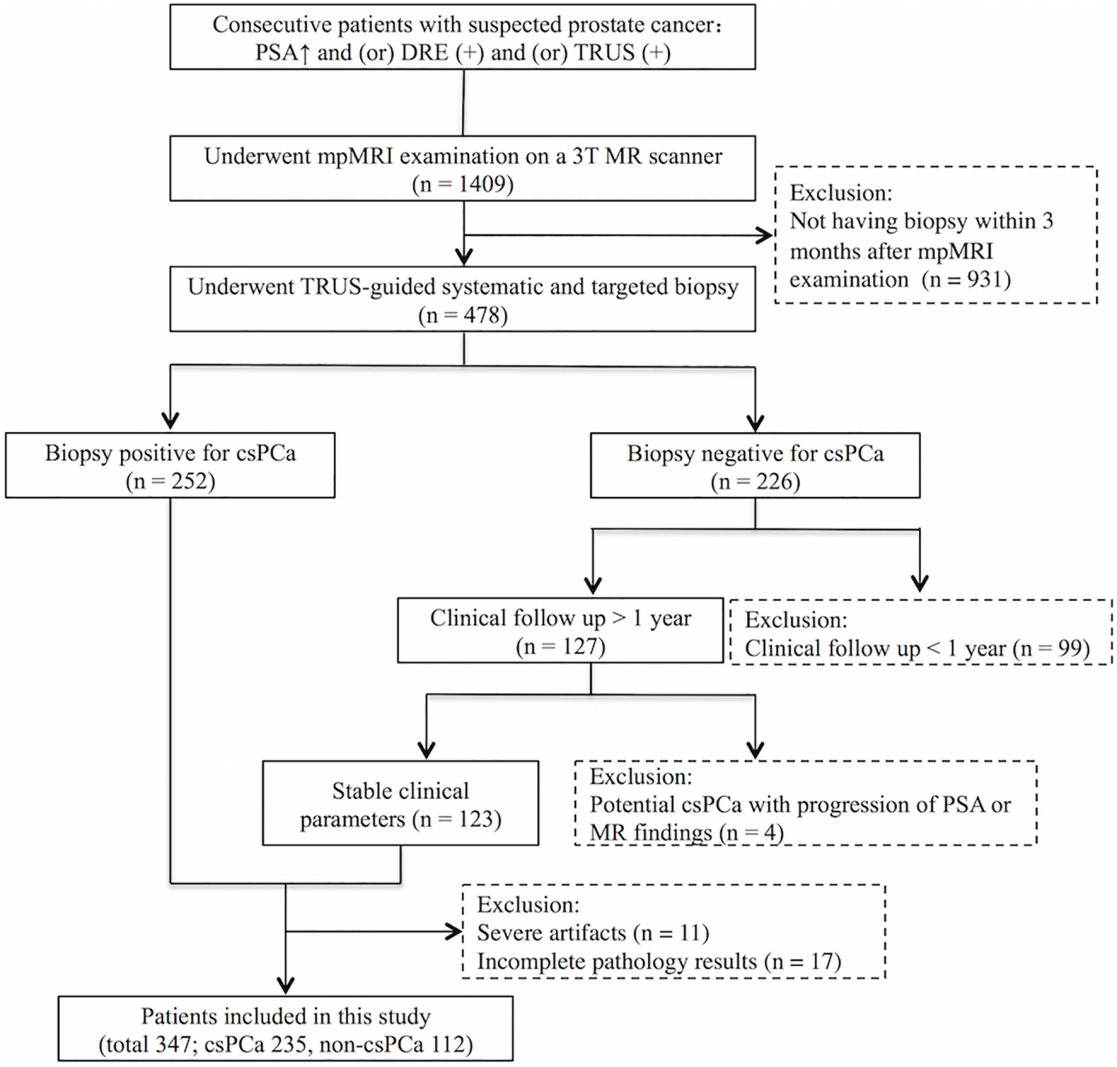
Flow diagram for inclusion of patients into the study.

Our institute’s review board approved this retrospective study and waived the need for informed consent. The inclusion criteria for the study were mpMRI scans performed on a GE 750 3.0T MRI scanner at Peking University First Hospital from March 2017 to February 2020 on consecutive patients who underwent mpMRI before a biopsy, with a clinical suspicion of PCa due to an elevated serum prostate-specific antigen (PSA) level, abnormal digital rectal examination (DRE), and/or abnormal transrectal ultrasound (TRUS) results. Exclusion criteria were patients without a subsequent biopsy performed within 3 months after mpMRI examination, a negative biopsy for csPCa without clinical follow-up >1 year, or showing potential csPCa during the clinical follow-up (progression of PSA or MR findings), as well as images with severe artifacts or incomplete pathology results which could not be matched with MR images. In total, 347 patients were included. **Figure 1** displays the flow diagram for the inclusion of patients in the study. In this study, csPCa was defined as the International Society of Urological Pathology Gleason grade group ≥2, i.e., Gleason Score ≥7. The characteristics of the 235 patients with csPCa included are shown in **Table 1**. The other 112 patients without csPCa (labeled non-csPCa with a mean age of 64.1 ± 7.5 years) had a median PSA level of 8.0 ng/ml, with an interquartile range of 6.6–13.1 ng/ml. The patients were randomly selected to populate the datasets for training (145 csPCa, 35 non-csPCa), validation (21 csPCa, 6 non-csPCa), and testing (69 csPCa, 71 non-csPCa).

**Table 1:** Characteristics of patients with csPCa.

Characteristics	Patients with csPCa (n = 235)
Mean age (years)	70.2 ± 8.6
Median PSA (ng/mL)	16.3 (9.7–32.6)
Per-patient maximum Gleason score	
3 + 4	76
4 + 3	59
3 + 5, 5 + 3, 4 + 4	46
4 + 5, 5 + 4	54
No. of csPCa lesions per patient	
One lesion	173
Two lesions	49
Three lesions	10
Four lesions	3
Zone distribution of csPCa lesions	
Peripheral zone	212
Transition zone	101

csPCa, clinically significant prostate cancer; PSA, serum prostate-specific antigen.

### MRI sequences

All of the mpMRI examinations were performed on a 3-T MR machine (Discovery MR750, GE Medical Systems). A 32-channel abdominal phased array coil was used as the receiving coil. All patients were scanned following the unified prostate mpMRI protocol. The main sequence parameters are summarized in **Table 2**. The ADC map was automatically generated by the MR vendor software based on diffusion-weighted imaging (DWI) data with different b values. Concerning the patients included in this study, anonymized images were exported in the Digital Imaging and Communication in Medicine (DICOM) format.

**Table 2:** The main sequence parameters in this study.

	T2WI	DWI	DCE
Field of view (mm)	240 × 240	240 × 240	260 × 260
Acquisition Matrix	320 × 256	96 × 96	320 × 192
Repetition time (ms)	3200	3000	4
Echo time (ms)	85	60	1.3
Flip angle (degrees)	111	90	13
Slice thickness (mm)	4	4	3
Additional information	…	b values: 0–1400 s/mm^2^	Temporal resolution = 13 s;18 phases

T2WI, T2-weighted imaging; DWI, diffusion-weighted imaging; DCE, dynamic contrast material enhancement.

### PI-RADS assessment

All of the mpMRI cases in the dataset of the test were retrospectively interpreted according to PI-RADS (version 2.1) by a urogenital radiologist with 10 years of experience in prostate MRI diagnosis. The radiologist was informed of the clinical information of the patients, such as age, biopsy history, PSA, etc., but was blinded to the pathology results and the previous MRI reports. The lesions detected were delineated on a prostate sector map.

### Reference standard and annotation

All of the patients in this study underwent TRUS-guided systematic and targeted biopsy using 12- or six-core needles. For cognitive targeting, lesions suspected of malignancy on mpMRI had been marked on a prostate sector map (6) using structured reports by five dedicated urogenital radiologists during the clinical routine. Before the biopsy, MR images would be reviewed by at least one urogenital radiologist and one urologist at a multidisciplinary meeting to ensure the accuracy of suspicious lesions localization. The urologists obtained additional needle cores (two- to five-core needles) for each of the suspicious lesions during the TRUS-guided biopsy. Histopathology analysis of each specimen was performed by a urogenital pathologist with 11 years of experience.

Two experienced urogenital radiologists (X and Y with 7 and 13 years of experience in prostate MRI diagnosis, respectively) retrospectively reviewed all csPCa cases and mapped the detailed pathology results of the csPCa foci to the MR images with consensus. The ground truth of the csPCa lesion segmentation was obtained using manual delineation by the urogenital radiologist (X), in consensus with and under the supervision of the other urogenital radiologist (Y), using the open-source segmentation software ITK-SNAP (version 3.6 2015; available at www.itksnap.org) (21). The format of ADC and T2WI was converted from DICOM to NIFTI. Three-dimensional volumes of interest (VOIs) were manually drawn along the boundaries of the csPCa lesions on consecutive axial sections of ADC images.

### Image preprocessing

After collecting the mpMRI data, the first step of image preprocessing is T2WI and DWI/ADC image registration. Patient motion is minimal and the two sequences are temporally close to each other during the scanning. T2WI and DWI/ADC images were registered *via* rigid transformation using the coordinate information stored in the DICOM image headers. B-spline interpolation to the third order was employed for all MR image interpolation tasks, while Gaussian label interpolation was used for the csPCa and prostate segmentation masks. Following this, a coarse segmentation of the prostate was obtained by K-means clustering in DWI high b value images to localize the prostate region. Once the prostate region was identified, the images were cropped to a patch of size 128 × 128. The prostate region of interest was then normalized into the range of [0, 1]. We augmented the data in the training set by mirroring, random rotation (rotation angle within 10°), and adding noise (within 0.001, which means each pixel value randomly fluctuates within one thousandth).

### Deep learning framework

**Figure 2:**
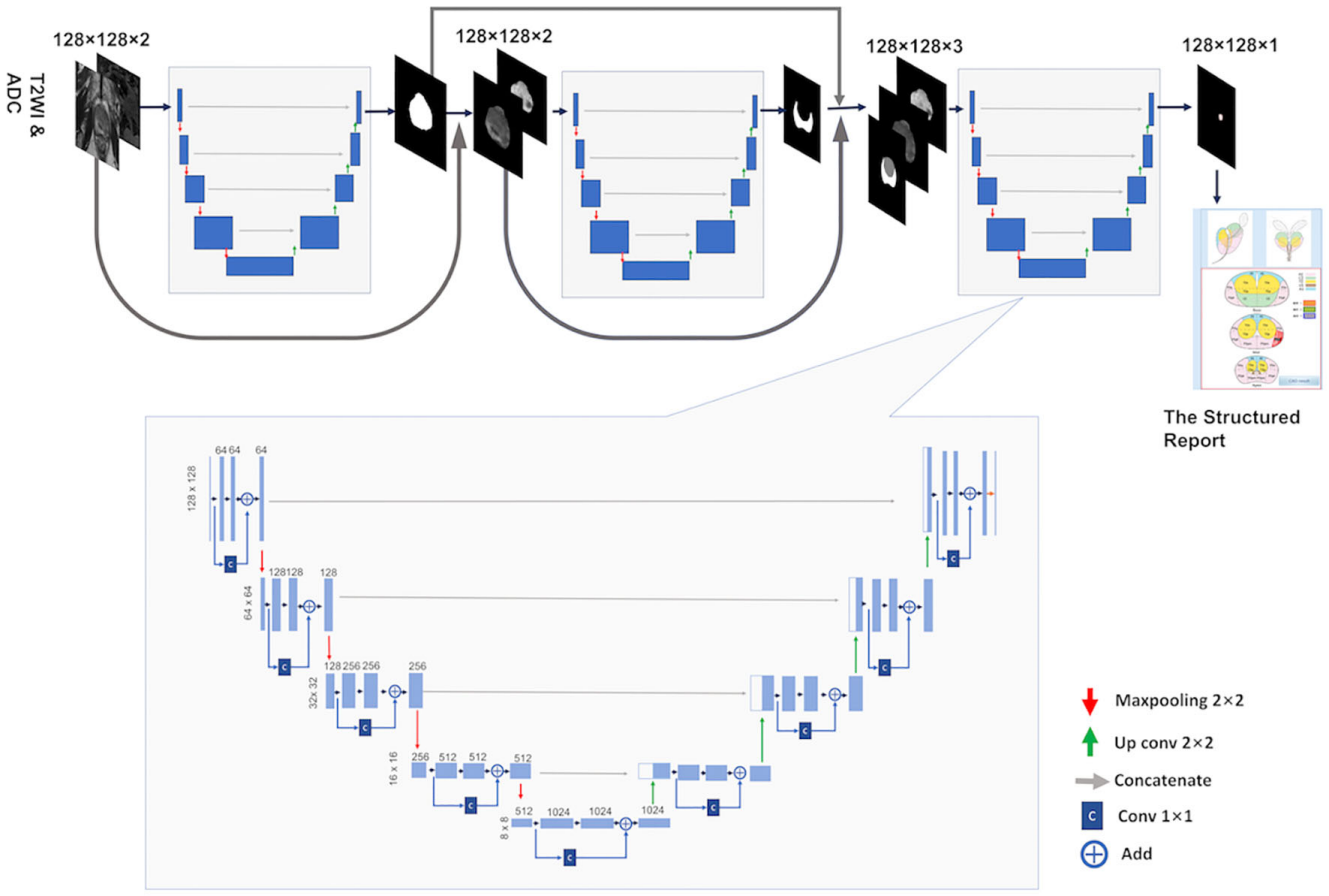
The briefarchitecture of the proposed weighted Res-Unet and the overall pipeline of our approach.

The base architecture for the deep learning framework used in this study is a CNN inspired by the 2D U-Net (22) and Res-Net (23) architectures and is termed Res-UNet. U-Net is one of the end-to-end methods of deep learning, which is also a pixel-to-pixel method and, with long skip connections, considers feature maps of the encoder path to obtain good segmentation performance in medical images. Res-Net (23) proposed a residual connection architecture to make the network deeper and avoid gradient vanishing and take advantage of strengths from both architectures by modifying the original U-Net architecture and adding residual blocks into the contracting and symmetric expanding paths of the U-Net architecture. In building the Res-UNet, we define a basic convolution operator by a 3 × 3 convolution (Conv) followed by a batch normalization (BN) and a rectified linear unit (ReLu). The residual block was designed by using a 1 × 1 Conv layer, plus an addition operation and ReLU function. **Figure 2** depicts the Res-UNet architecture.

Following McNeal’s criterion (24), the prostate is typically partitioned into two distinct zones: the Central Gland (CG, including both the transition zone and the central zone) and the Peripheral Zone (PZ). PCa lesions vary in frequency and malignancy depending on the zone, and there are different evaluation criteria for different regions in the PI-RADS. Therefore, just like a radiologist, a model for automated PCa detection and classification will invariably benefit from having both CG and PZ mask priors provided as inputs, in addition to the mpMRI. Accordingly, a cascading system of a three-segmentation Res-UNet which was previously developed in our institution (25) was developed for automatic prostate CG and PZ segmentation and PCa lesion segmentation. The cascade is designed to decompose the multi-task segmentation problem into a sequence of three smaller binary segmentation problems according to the subregion hierarchy. As can be seen in **Figure 2**, the first Res-UNet model takes prostate MR images (T2WI + ADC or only ADC) as inputs and produces a prostate segmentation mask as an output. Then, the second model takes the MR images and the prostate segmentation mask which were obtained in the previous step as inputs and produces a PZ segmentation mask. The CG segmentation mask can be computed by subtracting the PZ mask from the prostate mask. Finally, the last model takes the MR images, the PZ mask, and the CG mask as inputs and segments to the csPCa.

### Training setup

All of the training steps were performed using a GPU NVIDIA Tesla P100 16G at Peking University First Hospital, using the operating system Ubuntu 16.04. The software and packages used included Python 3.6, Pytorch 0.4.1, Opencv 3.4.0.12, Numpy 1.16.2, and SimpleITK 1.2.0. The input data were 128 × 128 images of ADC alone and ADC combined with T2WI, respectively, with an annotation of the csPCa lesions. The automated segmentation of the whole prostate gland and its different zones was completed using the previously developed and described method (25). For training the architectures for csPCa segmentation, the batch size was set as 20 with a learning rate of 0.0001. The networks were trained for 120 epochs. The pixel classification threshold was 0.5, while Adam was used as a training optimizer. The Dice similarity coefficient (DSC) was used to evaluate the performance of the networks in the segmentation of the csPCa, which is calculated as


$$\mathrm{DSC}=\frac{2/X\cap^{}Y/}{\;/X/+/Y/}$$

here X is the pixel set of csPCa segmented manually as the ground truth and Y is the pixel set of csPCa prediction by the model.

### Prediction results integrated into the structured report

Initially, the prediction results were “csPCa” or “non-csPCa” depending on the patient concerned. When “csPCa” was the output, the three-dimensional diameter of the suspicious csPCa lesions and the whole prostate gland would be filled into the structured report, as well as the key image of the suspicious csPCa lesions (**Supplementary Figure S1**). Furthermore, sextant localization of the suspicious csPCa lesions would be labeled in the prostate sector map. The prostate sextant is defined according to the standard sextant biopsy (26), based on the automatic segmentation of the prostate gland.

### Statistical analysis

Statistical analysis was carried out using SPSS 20.0 and MedCalc 15.8. We evaluated the performance of the biparametric (ADC + T2WI) model, ADC model, and PI-RADS assessment using the testing set. For PI-RADS assessment, PI-RADS ≥3 was considered positive for csPCa. For per-lesion analysis, to limit the influence of very small overlap regions, only the predicted lesions of the model overlapping ≥50% of the manual csPCa segmentation lesions in at least one slice were considered as true positive. Otherwise, the predicted lesions were considered to be false positives. For sextants analysis, only sextants overlapping at least 50% of an MRI lesion, or being occupied at least 50% by an MRI lesion, were considered to contain the MRI lesion (13). For per-patient analysis, if a patient had one or more than one csPCa lesions, the prediction of the model or PI-RADS assessment, was considered as true positive when at least one csPCa lesion was detected. On the other hand, for a patient without csPCa, the prediction was considered as false positive as long as one lesion was predicted. The performance of the models and PI-RADS assessment for csPCa detection and localization were evaluated based on the lesions, sextants, and patients, respectively. The sensitivity, specificity, and accuracy of the models and PI-RADS were evaluated and compared using the McNemar test. A p value of less than 0.05 was considered statistically significant.

## Results

### Based on lesions

In the test set, 88 csPCa lesions were included and the mean greatest dimension was 1.6 ± 0.7 cm. **Table 3** depicts the sensitivity of the models and PI-RADS assessment on the per-lesion analysis. The per-lesion sensitivity of the biparametric model, ADC model, and PI-RADS assessment was 95.5% (84/88), 94.3% (83/88), and 94.3% (83/88), respectively, with all p > 0.05. For the csPCa lesions with the greatest dimension ≥1.5cm, the sensitivity of the biparametric model was 100%, and the sensitivity of the ADC model and PI-RADS assessment was 97.6% (40/41, with p > 0.05). The sensitivity showed no significant difference between the models and PI-RADS regardless of whether the csPCa lesions were in the PZ or the TZ.

**Table 3:** Per-lesion sensitivity of the models and PI-RADS assessment.

	Biparametric model (%)	ADC model (%)	PI-RADS (%)	p
Total	95.5 (84/88) [88.8, 98.8]	94.3 (83/88) [87.2, 98.1]	94.3 (83/88) [87.2, 98.1]	>0.05
Peripheral zone	95.0 (57/60) [86.1, 99.0]	93.3 (56/60) [83.8, 98.2]	96.7 (58/60) [88.5, 99.6]	>0.05
Transition zone	96.4 (27/28) [81.7, 99.9]	96.4 (27/28) [81.7, 99.9]	89.3 (25/28) [71.8, 97.7]	>0.05
Dimension 0.4-1.5cm	91.5 (43/47) [79.6, 97.6]	91.5 (43/47) [79.6, 97.6]	91.5 (43/47) [79.6, 97.6]	>0.05
Dimension ≥1.5cm	100.0 (41/41) [96.4, 100]	97.6 (40/41) [87.1, 99.9]	97.6 (40/41) [87.1, 99.9]	>0.05

PI-RADS, Prostate Imaging Reporting and Data System.

**Figure 3:**
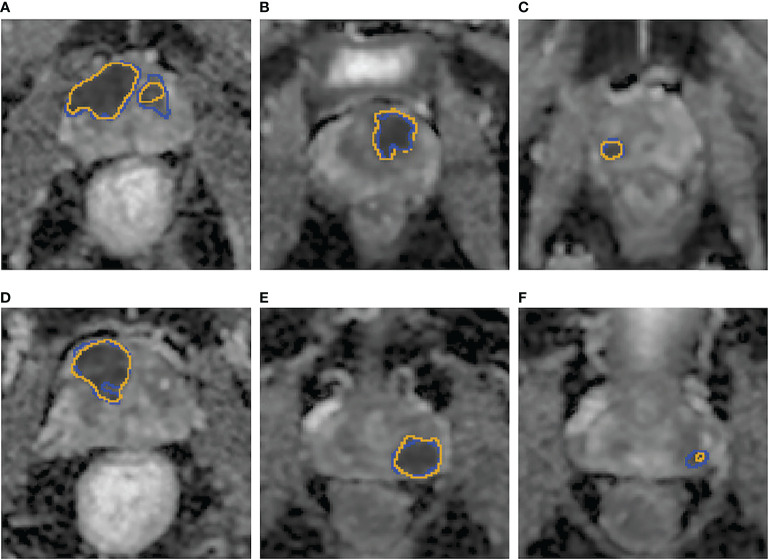
**(A–F)** Examples of the csPCa lesion segmentation performance of the biparametric model. The prediction results **(A–F)**, yellow line on the ADC map were highly consistent with the manual annotation **(A–F)**, blue line on ADC map by experienced urogenital radiologists according to pathological results.

In addition, the mean DSC based on csPCa lesions in the test were 0.64 ± 0.24 and 0.66 ± 0.23 for the biparametric model and the ADC model, respectively. **Figure 3** demonstrates examples of the csPCa segmentation of the biparametric model.

### Based on sextants

**Figure 4:**
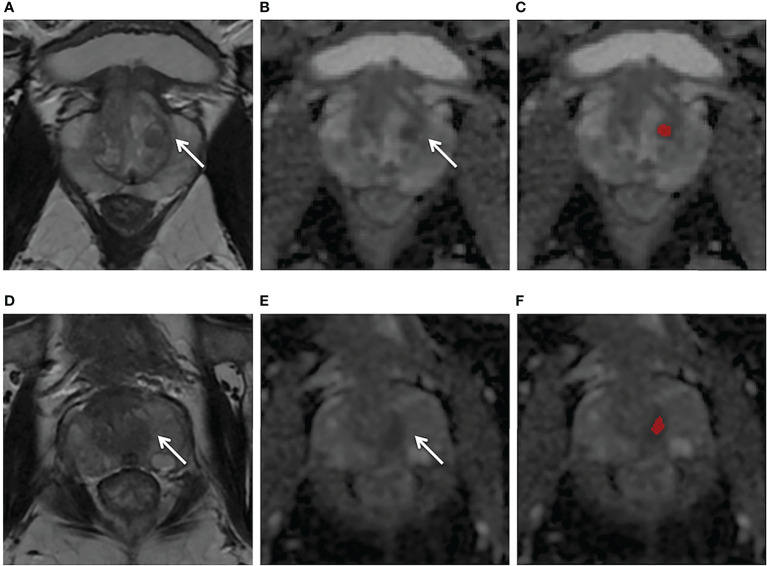
**(A–C)** Axial MR images obtained in a 56-year-old patient with a PSA level of 4.2 ng/ml and with negative biopsy findings. T2WI **(A)** showed a heterogeneous encapsulated nodule in the left transition zone (arrow) and the ADC map **(B)** showed hypointensity (arrow). The ADC model **(C)** appeared false positive (red region). **(D–F)** Axial MR images obtained in a 64-year-old patient with a PSA level of 5.9 ng/ml and with negative biopsy findings. T2W **(D)** and ADC **(E)** showed a normal left central zone, while the ADC model **(F)** appeared false positive in this area (red mark). The biparametric model gave negative predictive values for both cases.

A total of 840 sextants from the test set were analyzed, including 113 sextants of csPCa and 727 sextants of non-csPCa. The diagnostic efficacy and comparisons of the models and the PI-RADS assessment based on sextants are summarized in **Tables 4** and **5**. The biparametric model and PI-RADS assessment both had relatively high sensitivity, specificity and accuracy, i.e., 95.6% (108/113) *vs*. 92.9% (105/113), 91.5% (665/727) *vs*. 92.2% (670/727), and 92.0% (773/840) *vs*. 92.3% (775/840), respectively, all with p > 0.05. The ADC model demonstrated a comparable sensitivity of 91.2% (103/113) when compared with the biparametric model where p = 0.125, while the ADC model had a specificity of 86.8% (631/727) and accuracy of 87.4% (734/840), which were both lower than the biparametric model (all p< 0.001). Considering all the mpMRI sequences and detailed pathological results, 61.5% (59/96) of the false-positive sextants from the ADC model were hyperplastic nodules and asymmetric central zone, which is shown in **Figure 4**, while the ratio was 45.2% (28/62) for the biparametric model.

**Table 4:** Performance of the models and PI-RADS assessment based on sextants and patients.

	Sensitivity (%)	Specificity (%)	Accuracy (%)
Based on sextant			
Biparametric model	95.6 (108/113) [90.0, 98.6]	91.5 (665/727) [89.2, 93.4]	92.0 (773/840)
ADC model	91.2 (103/113) [84.3, 95.7]	86.8 (631/727) [84.1, 89.2]	87.4 (734/840)
PI-RADS	92.9 (105/113) [86.5, 96.9]	92.2 (670/727) [90.0, 94.0]	92.3 (775/840)
Based on patient			
Biparametric model	98.6 (68/69) [92.2, 99.9]	64.8 (46/71) [52.5, 75.8]	81.4 (114/140)
ADC model	97.1 (67/69) [89.9, 99.7]	54.9 (39/71) [42.7, 66.8]	75.7 (106/140)
PI-RADS	98.6 (68/69) [92.2, 99.9]	66.2 (47/71) [54.0, 77.0]	82.1 (115/140)

PI-RADS, Prostate Imaging Reporting and Data System.

Data in brackets are 95% CIs.

**Table 5:** Comparisons of the models and PI-RADS assessment based on sextants and patients.

	Sensitivity	Specificity	Accuracy
Based on sextants			
Biparametric model *vs*. ADC model	0.125	**<0.001**	**<0.001**
Biparametric model *vs*. PI-RADS	0.508	0.630	0.910
ADC model *vs*. PI-RADS	0.754	**<0.001**	**<0.001**
Based on patients			
Biparametric model *vs*. ADC model	1.000	0.118	0.077
Biparametric model *vs*. PI-RADS	1.000	1.000	1.000
ADC model *vs*. PI-RADS	1.000	0.077	0.064

PI-RADS, Prostate Imaging Reporting and Data System.

Bold characters indicate that the difference was statistically significant (p< 0.05).

### Based on patients

For the 140 patients (csPCa, 69; non-csPCa, 71) in the test set, the performance and comparisons of the models and the PI-RADS assessment based on patients are shown in **Tables 4** and **5**. Biparametric model and PI-RADS assessment had comparable per-patient sensitivity, specificity, and accuracy, i.e. 98.6% (68/69) *vs*. 98.6% (68/69), 64.8% (46/71) *vs*. 66.2% (47/71) and 81.4% (114/140) *vs*. 82.1% (115/140), respectively (all p > 0.05). ADC model had a similar sensitivity of 97.1% (67/69) compared with the combined model and PI-RADS assessment. The specificity and accuracy of the biparametric model were slightly higher than those of the ADC model (specificity, 54.9%; accuracy, 75.7%); however, the statistical significance was not reached (p = 0.118, 0.077).

## Discussion

Our approach using cascaded CNNs could automatically detect and segment the suspicious csPCa lesions on MR images without any human intervention. The whole prediction process could be completed within a few seconds per case, which was much faster than human interpretation using PI-RADS, which normally takes several minutes. There are several benefits of using a cascaded framework for the segmentation of hierarchical structures. First, many proposed methods try to solve the segmentation problem using a single neural network. Considering the great variability in the shape, size, texture, and appearance of the prostate gland and PCa, we suggest using cascaded CNNs for the segmentation task to improve the segmentation accuracy, and each network can focus on one segmentation problem. Thus, they are easier to train and can reduce over-fitting. Second, in consideration of the PCa lesions, these can vary in frequency and malignancy depending on the zone; the hierarchical pipeline follows the anatomical structures of the prostate and uses them as spatial constraints. Thus, the model for automated csPCa detection and classification will likely benefit.

The results demonstrated that the biparametric model had high sensitivity (95.5%, 95.6%, and 98.6% based on lesions, sextants, and patients respectively) and acceptable specificity (64.8%, 91.5%; based on patients and sextants) and had comparable performance to PI-RADS evaluation by an experienced radiologist, which is consistent with Schelb’s findings (13). The preliminary results of our study add to the evidence that fully automated deep learning models for csPCa detection have now even reached the level of an experienced radiologist (13, 27). Further prospective studies based on large consecutive data are needed for clinical validation. Furthermore, our model could also determine the boundary of csPCa precisely. The DSC based on csPCa lesions was 0.64 and 0.66 for the biparametric model and ADC model, respectively, which was higher than that in similar studies on csPCa detection that reported 0.35–0.58 (13, 28, 29). The good segmentation performance would facilitate the 3D prostate MRI-TRUS fusion targeted biopsy. Additionally, a 3D model for the visualization of csPCa and the adjacent vital structures, based on accurate segmentation, may be helpful for the urologist in the surgery, as well as for patient education (30). However, DSC as a voxel-level metric remains limited for lesion-level PCa detection and can misrepresent the accuracy for evaluating the localization of multifocal PCa (31, 32). Therefore, our study based on the actual clinical practice mainly used sensitivity and specificity at the lesion, sextant, and patient levels to comprehensively evaluate the performance of the model.

T2WI and ADC derived from DWI are recommended by PI-RADS as the most important sequences for the evaluation of TZ and PZ lesions, respectively. Many studies demonstrated that the diagnostic performance of biparametric MRI without DCE was similar to those of mpMRI (33, 34). Therefore, this study mainly used those two parameters to develop the model. Additionally, the ADC of DWI is considered to be the current best monoparametric sequence of prostate MRI assessment, which is reported to have a strong relationship with the Gleason scores (GS) of PCa and is even superior to TRUS-guided prostate biopsy for the assessment of PCa aggressiveness (35–37). For this reason, the proposed model trained with monoparametric ADC was evaluated specifically. The results of our study demonstrated that the monoparameter ADC model had a high sensitivity for csPCa detection (94.3% [83/88] and 97.1% [67/69], based on lesions and patients, respectively), which showed no significant difference (p > 0.05) with the biparametric model and PI-RADS assessment, regardless of whether the csPCa lesions were located on the PZ or TZ. Zabihollahy et al.’s study also showed that deep learning using only ADC was highly sensitive and could even reach a 100% sensitivity at the level of dominant PZ tumor detection (12), which is slightly higher than ours. The reason may be that their study only considered the most dominant lesion on PZ, which was easily identifiable, while our study detected all the csPCa on the MR images. Further research with larger volumes of testing data is needed to verify the performance of the ADC model. The specificity of the biparametric model in our study was higher than that of the ADC model based on sextants (p< 0.001). When compared with the ADC model, the biparametric model outputted fewer false-positive lesions such as hyperplastic nodules and the central zone. Nevertheless, the high sensitivity of the ADC model using a single parameter instead of time-consuming mpMRI may facilitate the promotion of prostate MRI screening.

In contrast with studies that were training models using public data (15, 38), the data in our study were collected consecutively based on real-world clinical scenarios, which would allow the model to be more easily integrated into a clinical setting. The amount of data in the training and validation sets (145 and 21 csPCa cases, including 1204 slices and 173 slices, respectively, after automatic prostate segmentation) was comparable to that used in some studies (11, 12), and was larger than that in other studies (19, 20). Therefore, we put more data in the testing set (69 csPCa, 71 non-csPCa cases) to better evaluate and verify the generalization ability of the model. Our proposed biparametric model yielded high sensitivity of 98.6% for csPCa detection based on patients, as well as other studies with the sensitivity ranging from 82.9% to 97% (13, 14, 39–41). However, it is worth noting that the specificity was not as high as expected in our study (64.8%) and in other studies (47%–76%) (12–14, 39). That is to say, the success of the AI came at the cost of a high false-positive rate of even 50% (42). Yu et al. (43)proposed a cascaded approach to reduce the false positive for PCa detection, where the second-stage classifier was able to reduce false positives at the expense of nearly an 8% decrease in detection sensitivity. Saha et al. (29) present a multi-stage 3D CAD model for csPCa localization in biparametric MRI with the addition of a residual patch-wise 3D classifier to improve the model specificity. The results demonstrated that up to 12.89% less false positives were generated per patient, while retaining the same sensitivity (92.29%) as before. Min et al. (44) explored the feasibility of controlling the false positives/negatives during training by incorporating the cost-sensitive classification losses. More studies are needed to further explore how to improve the specificity of the prostate CAD.

An optimal AI model should not only have good performance for csPCa diagnosis but should also have a perfect output form facilitating clinical practice. One advantage of the proposed approach is that it had a perfect output and added AI into the radiological workflow seamlessly by automatically integrating the prediction results into structured reports, which makes this approach more convenient for clinical application. At present, our model can output whether a patient has csPCa or not and the size of the prostate gland. If csPCa lesions were found, it would further output the size of csPCa lesions and also mark the area of csPCa on MR images. These prediction results could be automatically transferred into a structured report before radiologists open the reporting system. However, a complete structured report includes many other contents, such as whether the csPCa lesions invade the prostate capsule and adjacent structures, as well as lymph node condition, bone metastasis, etc. Our institution is now exploring each of the above, and some of them have achieved good performance (45). Our ultimate goal was to develop a fully automatic intelligent structured report, thereby freeing radiologists from heavy clinical paperwork. When radiologists open structured reports, they just need to check the accuracy of each item.

Several limitations of our study were as follows. First, all of the images were from a single MR machine in a single institution. Multi-center and multi-machine data functionality should be added to improve the generalization ability of the model in further studies. Secondly, this U-Net model was trained only using ADC and T2WI. Future research involving the addition of more MRI sequences and/or clinical information may be investigated to improve the performance of the model. On the other hand, the model in this study achieved good results with biparametric MRI, so streamlining MRI sequences with an advanced algorithm may be another possible research direction. Thirdly, even though the reference standard using TRUS-guided systematic and targeted biopsy had high sensitivity for csPCa, it still has a false-negative rate when compared with radical prostatectomy. Nevertheless, our cohorts may be optimal, for radical prostatectomy cohorts would exclude many patients who only had a prostate biopsy and could lead to bias. Finally, this model was only applicable to the detection and localization of csPCa instead of staging and active surveillance.

## Conclusion

In conclusion, our study demonstrated that a cascaded deep learning model trained with ADC and T2WI achieved good performance for the fully automated detection and segmentation of csPCa and demonstrated comparable performance with an experienced radiologist using PI-RADS (version 2.1). The proposed approach can automatically integrate prediction results into the radiological workflow seamlessly by using a structured report. As a preliminary exploration, this study provided a reference for future AI clinical implementation. Further studies are needed to explore the optimal paradigm of AI clinical integration.

## Data availability statement

The datasets presented in this article are not readily available because the datasets are privately owned by Peking University First Hospital and are not made public. Requests to access the datasets should be directed to XYW, wangxiaoying@bjmu.edu.cn.

## Ethics statement

The studies involving human participants were reviewed and approved by Peking University First Hospital. Written informed consent for participation was not required for this study in accordance with the national legislation and the institutional requirements.

## Author contributions

LZ and XYW designed the study. CH, XL, and DL contributed to acquisition of data. LZ and XYW annotated the images data. YZ, WL, XPW, and JZ designed the model and implemented the main algorithm. LZ and GG analyzed the data. LZ wrote the paper. XYW and XZ reviewed the paper. All authors contributed to the article and approved the submitted version.

## Acknowledgments

The authors would like to acknowledge Xin Yue and Suisui Zhang from the Beijing Smart Tree Medical Technology Co., Ltd., for their help in constructing the structured report.

## Conflict of interest

Authors WL, XPW and JZ are employed by Beijing Smart Tree Medical Technology Co. Ltd.

The remaining authors declare that the research was conducted in the absence of any commercial or financial relationships that could be construed as a potential conflict of interest.

## Publisher’s note

All claims expressed in this article are solely those of the authors and do not necessarily represent those of their affiliated organizations, or those of the publisher, the editors and the reviewers. Any product that may be evaluated in this article, or claim that may be made by its manufacturer, is not guaranteed or endorsed by the publisher.
