# Supplementary figures and images for: Fully automated detection and localization of clinically significant prostate cancer on MR images using a cascaded convolutional neural network
Source: Front Oncol. 2022 Sep 29;12:958065. doi: 10.3389/fonc.2022.958065 (PMC9558117; doi:10.3389/fonc.2022.958065)

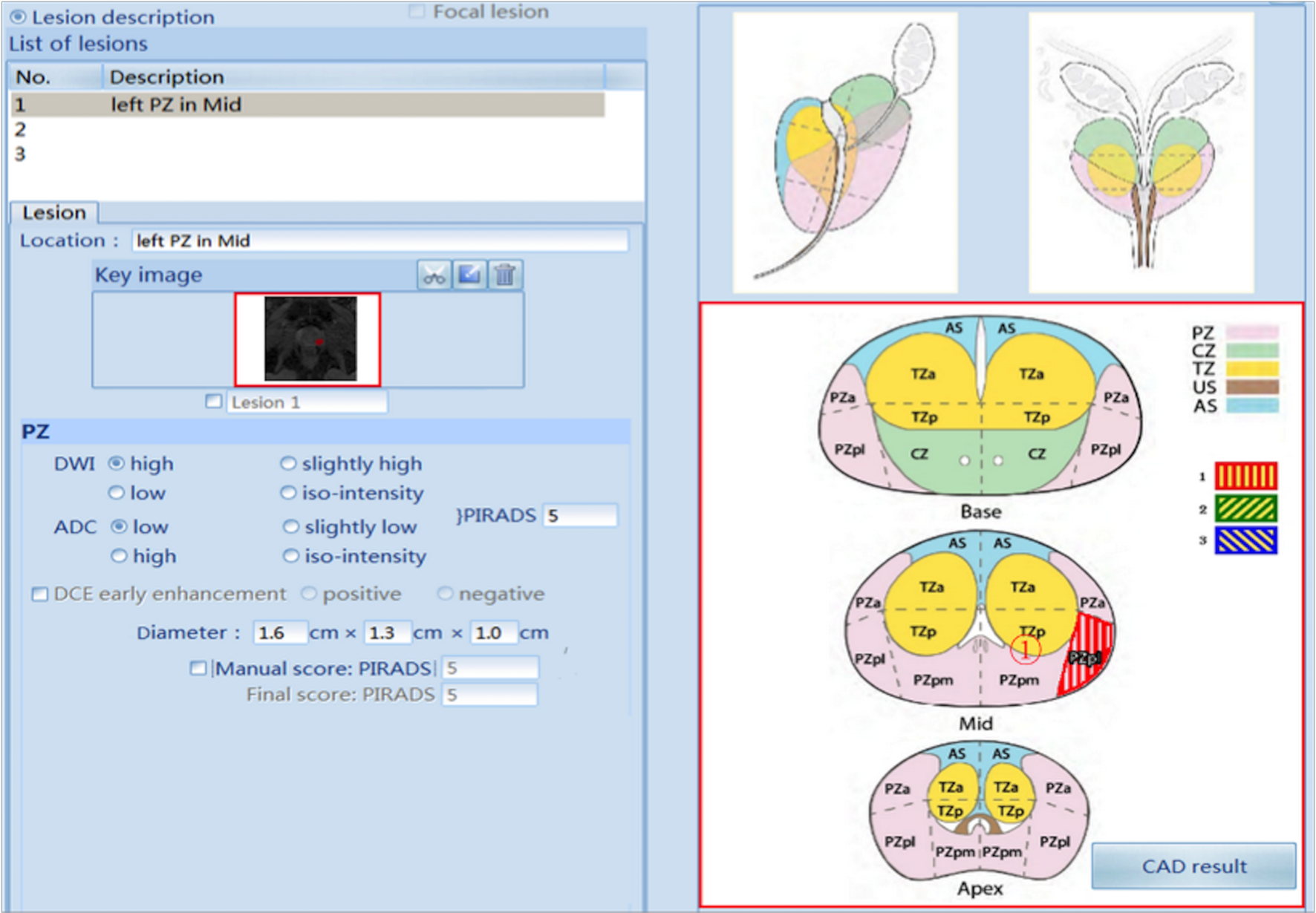

Supplement: Supplementary file 1 [file Image_1.tiff]
